# Using donor human milk to feed vulnerable term infants: a case series in KwaZulu Natal, South Africa

**DOI:** 10.1186/s13006-018-0185-6

**Published:** 2018-09-10

**Authors:** Penelope Reimers, Natalie Shenker, Gillian Weaver, Anna Coutsoudis

**Affiliations:** 1 Department of Paediatrics and Child Health, University of KwaZulu-Natal, 719 Umbilo Rd, Durban, 4001 South Africa; 2 Hearts Milk Bank, Biopark, Broadwater Road, Welwyn Garden City, AL7 3AX UK; 3 Imperial College London, Du Cane Road, W12 0NN, London, UK

**Keywords:** Breastfeeding, Case series, Donor human milk, Fullterm infants, Failure to thrive, HIV

## Abstract

**Background:**

Donor human milk is the World Health Organization’s recommendation for infant feeding when the mother’s own breast milk is unavailable. Breast milk has been shown to reduce morbidity and mortality and in low birthweight infants, donor milk reduces the incidence of necrotising enterocolitis, late onset sepsis and improves outcomes. There is a paucity of literature documenting outcomes of using donor human milk in older children who need additional support for a variety of health issues.

**Case presentation:**

A series of seven case studies is presented of orphaned and abandoned children, many of whom were either HIV exposed or positive. All children were fed with pasteurised donor human milk at a transition home and their progress reported.

**Conclusions:**

Although detailed medical records were not always available, the case studies provide anecdotal evidence of the protective effects of donor human milk against failure to thrive, diarrhoea, atopic dermatitis, and opportunistic infections.

## Background

*The Lancet* Breastfeeding Series (2016) presented compelling evidence regarding the short and long-term health implications of breastfeeding and its critical role in reducing mortality and morbidity in children and mothers [1]. These findings were evident in low-, middle- and high-income countries, but varied regionally with impacts on both infectious and non-communicable disease.

The World Health Organization recommends that the first option when a mother is unable to breastfeed her child should be the use of donor human milk (DHM) [2]. This recommendation is supported by guidelines published by the American Association of Pediatrics [3] and the European Society for Paediatric Hepatology, Gastroenterology and Nutrition [4]. Donor human milk is defined as breast milk that is expressed by a mother and processed by a human milk bank for use by a recipient that is not the mother’s own baby. Over the past decade, research has established multiple benefits of the use of DHM for low birthweight infants in neonatal intensive care units when maternal milk is not sufficiently available. These include a reduction in rates of necrotising enterocolitis and late-onset sepsis, and improved cardiovascular outcomes [5–8]. Donor human milk has also been associated with reduced durations of hospital stay and improved maternal breastfeeding rates in the neonatal intensive care units [9, 10]. There is a paucity of literature documenting the use of DHM for full-term and older infants with additional health needs, although anecdotal evidence has suggested applicability in a range of conditions where maternal milk is unavailable.

There are seven million people living with HIV/AIDS in South Africa [11] and 3.7 million orphans [12]. Approximately 3,500 babies are abandoned each year and these numbers are increasing [13]. In 2001, the first community based human milk bank in South Africa was established by one of the authors to meet the needs of abandoned and orphaned infants cared for in a transition home, iThemba Lethu [14]. In over 16 years of operation, 93 babies and children have been adopted into families and 26 have been reunited with family. Of these 62 infants who ranged in age from birth to three years, both HIV-exposed and non-exposed, were fed with screened, pasteurised DHM during their stay in the home. Over 3 000 l of DHM were collected during this time, and donor mothers were recruited from the surrounding community through Child Health Clinics and hospitals.

We present a series of representative cases of infants who received screened DHM at iThemba Lethu (ITL) for nutrition as a routine part of their care, to illustrate that human milk, despite being previously pasteurised and frozen, offers a practical and safe source of nutrition, and provides protection against infections and support for normal infant development, and an alternative to infant formula when maternal milk is unavailable.

## Case presentations

Detailed medical histories of many of the infants placed in care at ITL were often unavailable due to poor socioeconomic circumstances and frequent abandonment. Recent improvements in the prevention of mother-to-child transmission by the treatment of HIV positive women has resulted in fewer infants who are HIV positive at birth. This was not the case when the community milk bank began operating. Some of the case reports date back to 2003 and 2006, but have been included as these infants were severely compromised on arrival at ITL and together with medical attention, the progress they made on DHM was marked. Given the paucity of documented case histories of exclusive DHM nutrition in a non-hospital based and older infants, their inclusion is important.

The routine medical management of HIV positive infants at ITL included screening for tuberculosis and prophylactic treatment with co-trimoxazole which was started at four to six weeks after birth and continued until they were five years of age. In addition, they received antiretroviral treatment (ARV) when it became available. In the early days of treating HIV positive children, early routine ARV treatment was not practiced. Medical management was provided by a local teaching hospital. Scabies was treated with tetmosol soap and benzyl benzoate lotion and eczema with aqueous cream and different emollients. Tuberculosis was treated with an intensive 2-month phase of rifampicin/ isoniazid, pyrazinamide with lower-dose therapy using the same agents, for a further four months.

### Case 1

**Fig. 1:**
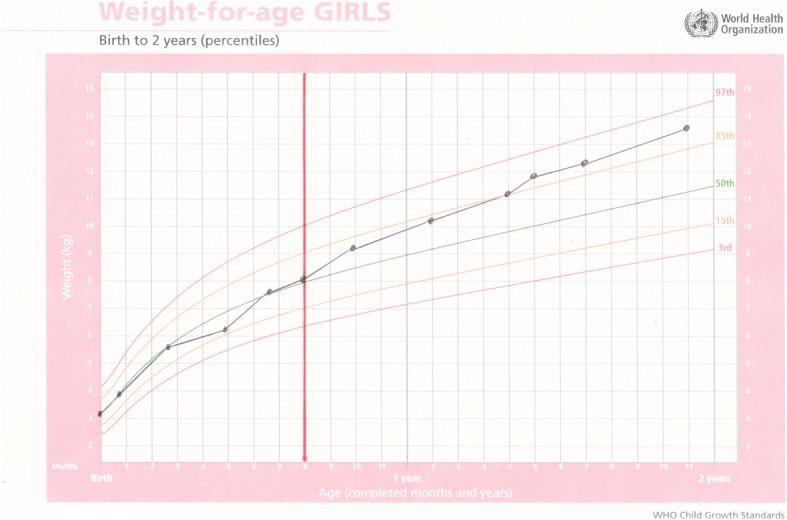
Weight for age chart for Baby A; the dark solid vertical line denotes intervention with DHM

Baby A was born at term by caesarean section due to fetal distress. Her birthweight was 3.1 kg, length (L) 45 cm, head circumference (HC) 36 cm. The mother was HIV positive. Baby A was abandoned at six weeks, with a bag of clothing and a tin of infant formula and her birth record. Unable to trace the mother, she was placed in care and arrived at ITL on 21 September 2006 when she was eight months old. She weighed 8.18 kg, was HIV positive, Mantoux positive and negative for syphilis. Her eczema was severe, as is commonly associated with HIV positive children. She was started on DHM immediately. She was placed on treatment for tuberculosis from October 2006 until May 2007. In June 2007, her CD4 count was 18 and she was started on ARV. She had a severe dose of chicken pox and mumps at two years of age, but other than that had no repeated infections commonly associated with HIV positive children. Her milestones were appropriate for her age. She continued to receive DHM until she was three years old. The decisions were made to continue for a longer period than normal as whenever the DHM was stopped, her eczema was exacerbated. She was a happy, confident, intelligent little girl who enjoyed good health despite her medical status and ongoing treatment. She was adopted in July 2011 aged five years (Fig. 1).

### Case 2

Baby B was born weighing 2.5 kg. His mother and grandmother had full blown AIDS and he was diagnosed with HIV at birth. He was found in his home neglected, as his mother was too ill to care for him and she signed him over for adoption. He arrived at ITL at 2.5 months, weighing just 3.1 kg. He was suffering from malnutrition, tuberculosis, respiratory distress and was HIV positive. In addition, he had scabies and severe eczema. DHM was started upon arrival. A month later, at 3.5 months, he began treatment for TB. Apart from drainage of a few skin abscesses at four months of age, his general condition improved on the DHM, as did his eczema and he thrived and attained all his milestones appropriately. He remained on the DHM until he was 14 months old. At the age of 21 months he was started on ARVs which increased his CD 4 count from 13 to 30% in a six month period and was adopted at this time.

### Case 3

**Fig. 2:**
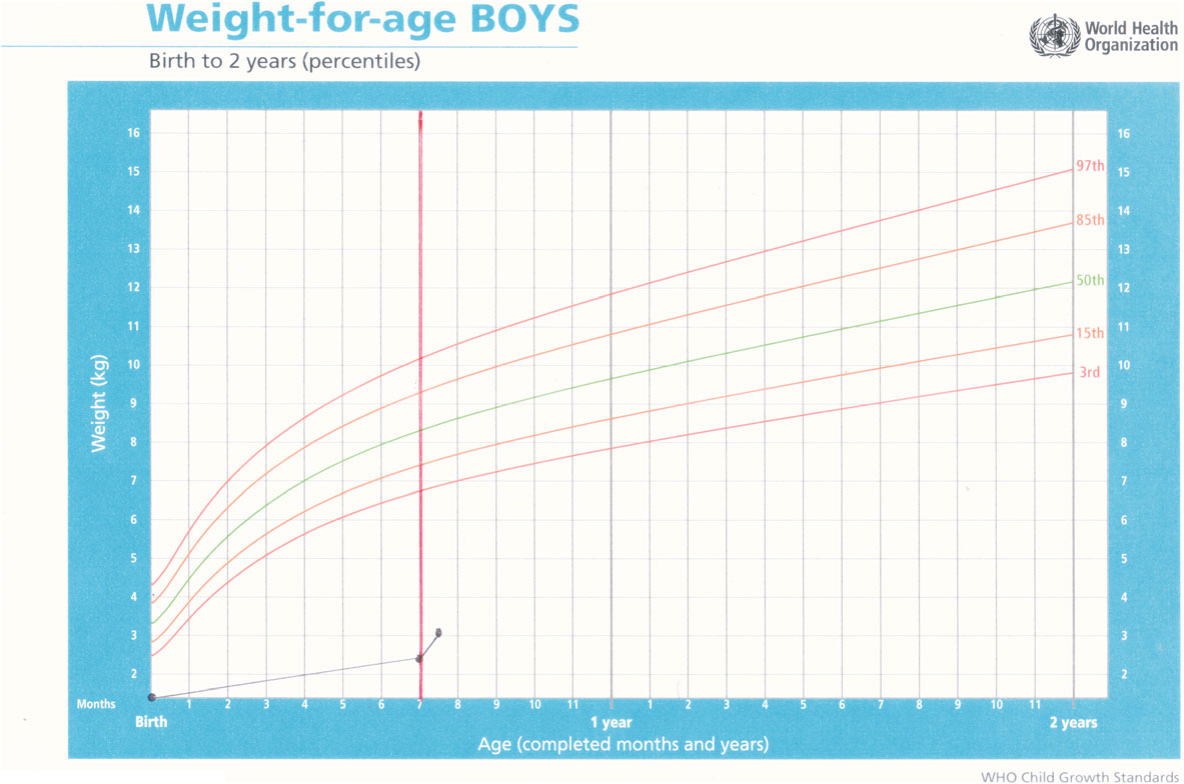
Weight-for-age chart for Baby C with rapid weight gain after commencing donor milk; the dark solid vertical line denotes intervention with DHM

Baby C was a male born preterm. There are no records of gestation and he weighed 1,500 g. The mother was 25 years old, HIV positive and abandoned the infant in hospital where he was also diagnosed as being HIV positive. He was placed in care on co-trimoxazole (sulfamethoxazole and trimethoprim) and antiretroviral prophylaxis. He failed to thrive. At five months he was admitted to hospital critically ill. An ultrasound of his head revealed prominence of ventricle and cerebrospinal fluid space which suggested brain atrophy. Due to his poor prognosis no ARVs were recommended. He was transferred to ITL at seven months of age, weighing 2.7 kg. He was immediately started on DHM and after two weeks of exclusive DHM he had gained 550 g. In seven months of exclusive formula feeding, he had gained only 1200 g. He continued to gain weight but at eight months was transferred to a hospice for nursing care as he was too ill to be placed in foster care or to be adopted and died a short while later (Fig. 2).

### Case 4

Baby D was admitted to a hospital in Durban, South Africa at two months with severe gastroenteritis, dehydration and marasmus. At approximately four months, he was still in hospital, he weighed 3.9 kg, the diarrhea had not improved and he had not gained weight in hospital despite intensive nutritional intervention. The infant was brought to ITL during a hospital workers strike, by medical officers at the hospital. He was not tolerating the specialized formula brought by the doctors, so he was offered DHM. During his two week stay there was an improvement in his condition. The diarrhea resolved, he started gaining weight, he started smiling and responding. Unfortunately, as he was not an infant placed in the care of ITL no records were kept of his weight gain during his time in our care. The two medical officers who collected him after the resolution of the strike made the following comments:

“*Improvement was radical, dermatitis had resolved, gastroenteritis resolved, child was more alert and responsive, even the fisting had improved somewhat. This study was retrospective and there are poor records but if medical science can be considered both a science and an art, what breastmilk did for that baby is a masterpiece*.” Medical Officers.

### Case 5

**Fig. 3:**
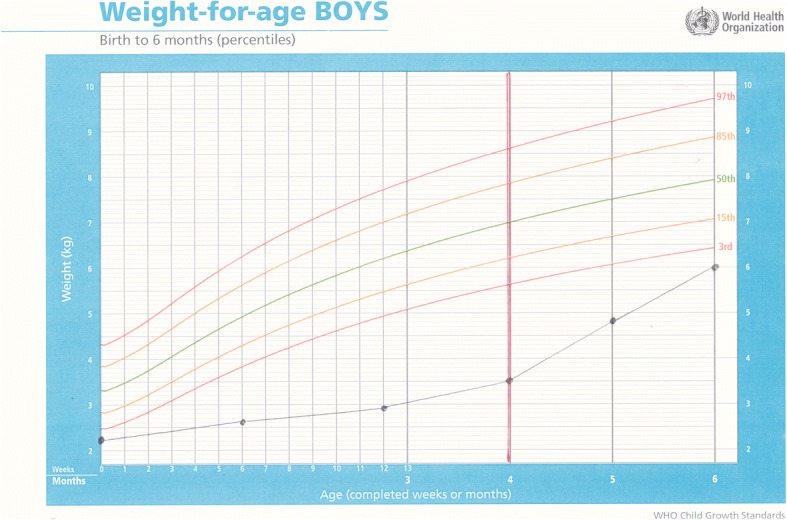
Weight-for-age chart for Baby E; showing poor weight gain prior to admission and dark solid vertical line denoting rapid weight gain after commencement on donor milk

**Fig. 4:**
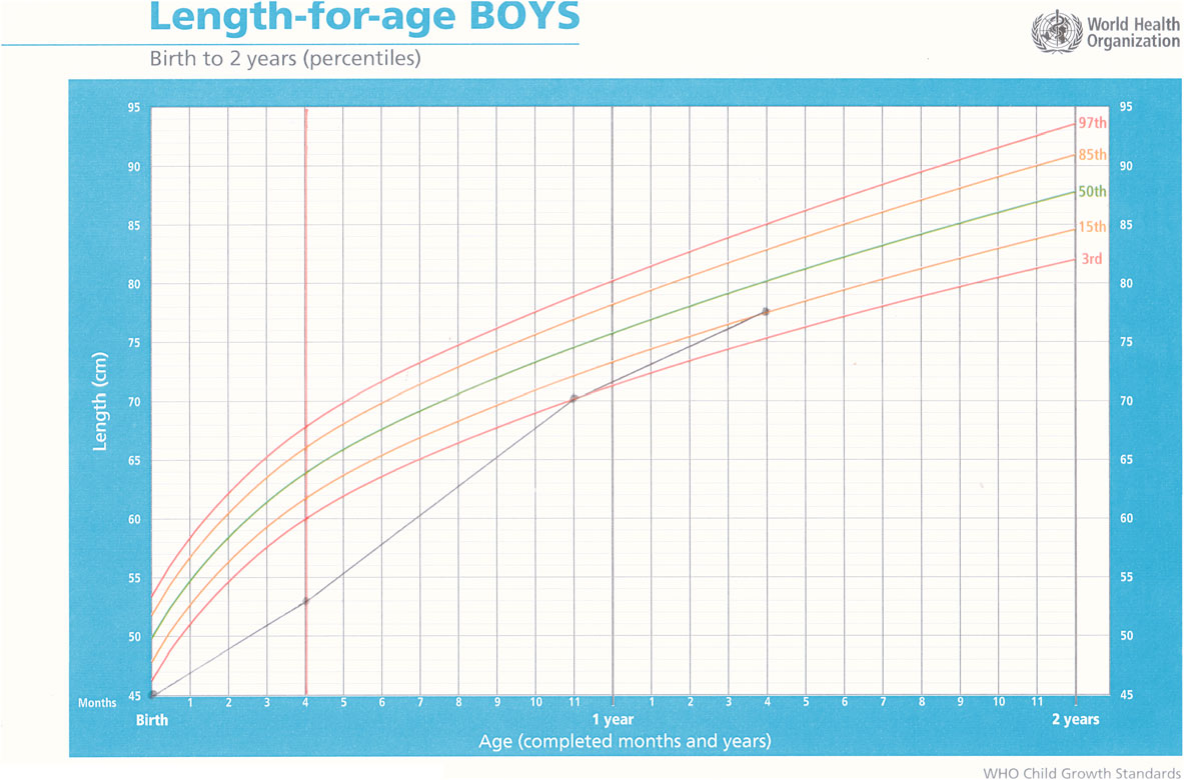
Length-for-age chart for Baby E; solid vertical line showing rapid improvement in length after admission

Baby E was born by normal vaginal delivery at 33 weeks gestation. He was twin two, with a birthweight of 2.250 g, Apgar 6/10, 8/10, L 44 cm, and HC 33 cm. His mother was 24 years old and HIV negative. Both infants were abandoned in the care of a neighbour when they were three months old. Baby E was admitted to hospital with severe malnutrition soon after weighing 2.9 kg, with bipedal oedema and a haemoglobin count of 7.5. On 15 August 2016 at four months of age, weighing 3.5 kg, L 53 cm and HC 39 cm, he was transferred to ITL and started on DHM immediately. On examination, he was found to have low set ears, epicanthic folds and a broad nose base, so was referred for genetic testing. His weight gain from birth had been poor. After a month on DHM, he had gained 1.3 kg, the same amount he had gained in the previous four months. Good weight gain continued on DHM, with complimentary food added at six months and DHM continued until he was eight months old. His genetic testing was normal and bloods taken for HIV, syphilis and hepatitis B were all negative. Milestones were somewhat delayed, he rolled over at six months, sat at nine months started crawling at a year and at 16 months was pulling himself up and walking around furniture. He is still being cared for at ITL (Figs. 3 and 4).

### Case 6

**Fig. 5:**
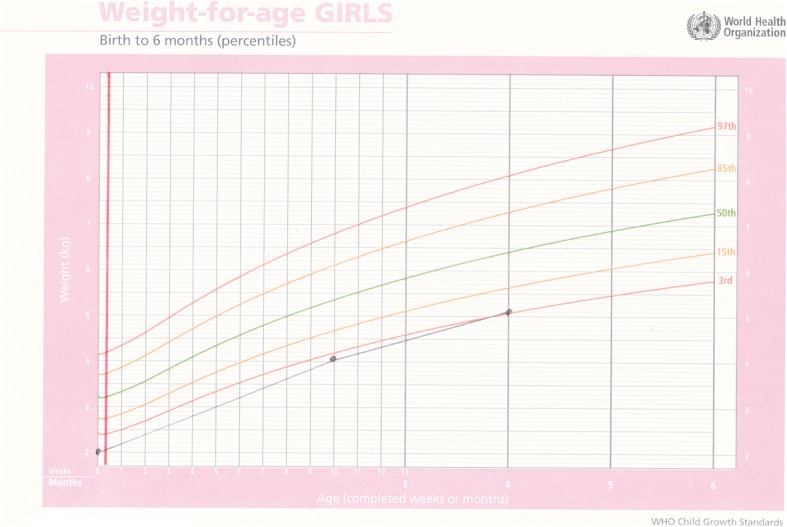
Weight-for-age chart for Baby F; the dark solid vertical line denotes intervention with DHM

**Fig. 6:**
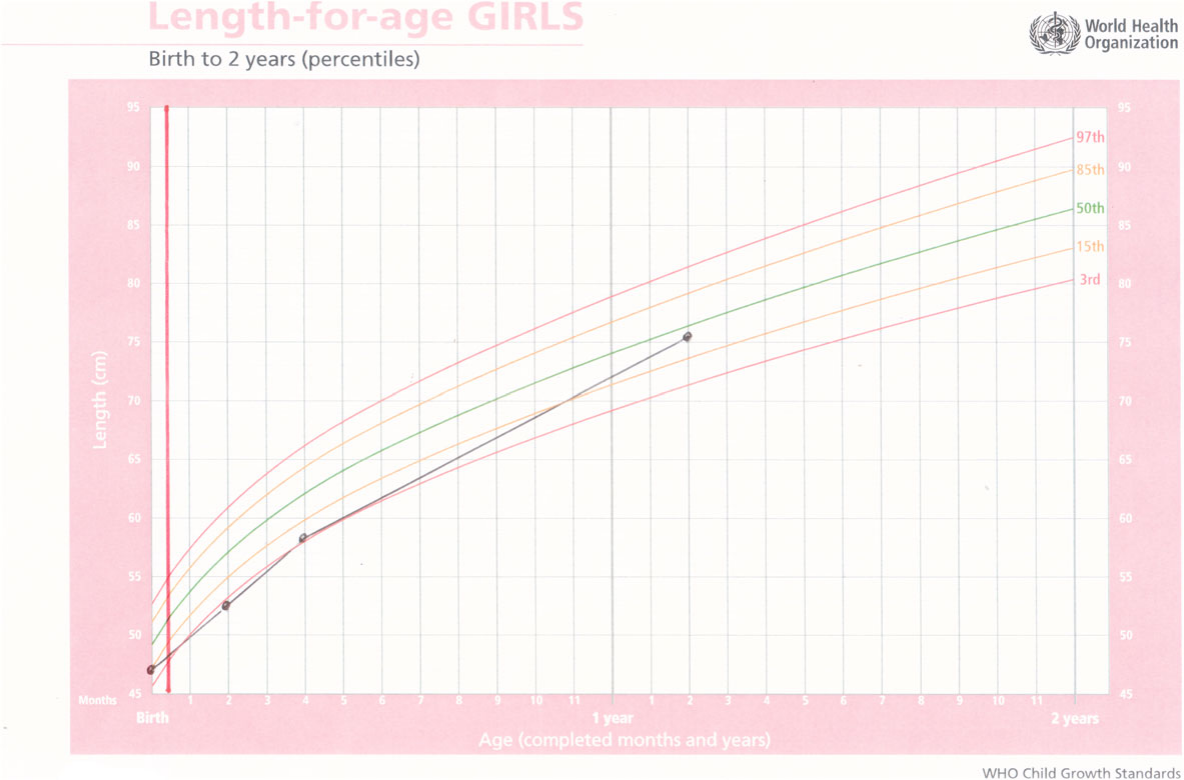
Length for age chart for Baby F; the dark solid vertical line denotes intervention with DHM

Baby F was a female born by an emergency caesarean section for fetal distress. Although term, her birthweight was 2 kg, L 47 cm, head circumference 34 cm. The mother was an HIV positive teenager. The infant was exclusively formula fed for 10 days and given nevirapine syrup as ARV prophylaxis for six weeks. She arrived at ITL on 14 August 2014 when she was 10 days old and was started on DHM immediately. All blood tests for HIV, syphilis and hepatitis B were negative. At three months, she weighed 5 kg, with L 57 cm and HC 40.5 cm. She was fed with DHM until her time of adoption at six months Figs. (Fig. 5 and 6).

### Case 7

**Fig. 7:**
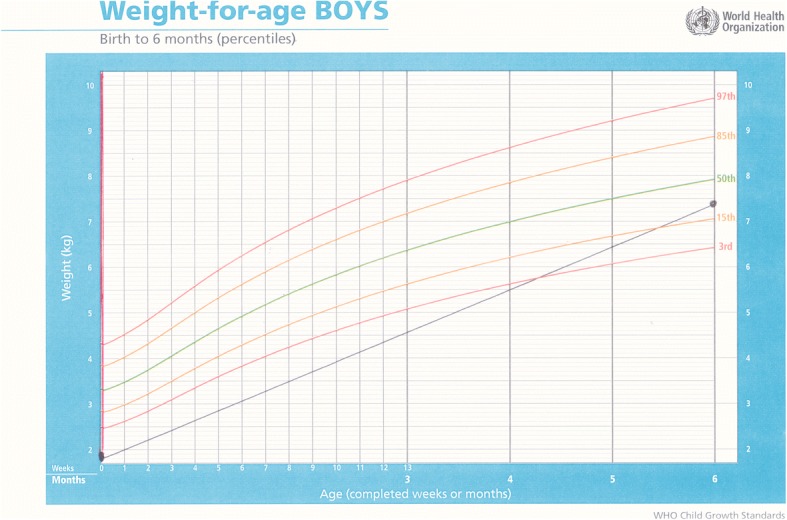
Weight for age chart for Baby G, who was commenced on DHM at birth

Baby G arrived at ITL in 2015 when he was one-day-old weighing 1.9 kg. His mother had concealed her pregnancy. The baby boy was fed with DHM from the day of arrival. He was switched to infant formula in November and December 2015, when supplies of DHM ran out; however, he did not tolerate the artificial substitute well and began vomiting after feeds. When DHM was available again in January 2016, his condition improved, the vomiting stopped and he gained weight once again. He was adopted at six months weighing 7.4 kg (Fig. 7).

## Discussion

To date there are few documented cases of infants outside of a hospital setting receiving donor milk and these case studies discussed above importantly provide a sizable body of anecdotal evidence of the protective effects of DHM against failure to thrive, diarrhoea, atopic dermatitis and opportunistic infections. The health benefits conferred by the breast milk are not surprising. Breast milk is known to provide optimal nutrition for growth and development. Donor human milk proved to be the personal medication for these infants, stimulating their often compromised immune systems. In 16 years of operation of ITL, there has not been a single adverse reaction to DHM, or a need to switch the source of nutrition to infant formula.

Horta and Victoras’ extensive review of the evidence suggests that breastfeeding substantially protects against morbidity/mortality from diarrhoea. This protection is highest in exclusively breastfed infants [15–17]. Rollins et al. [18] confirmed these findings and reported the frequency and length of diarrhoeal days were significantly reduced in EBF infants irrespective of if HIV exposed or not. A systematic review of literature (1980–2009) found a considerable body of evidence supporting the protective effects of breastfeeding against diarrhoeal incidence, prevalence and mortality [19].

Factors protective against gastro-intestinal infections include antimicrobial factors such as lactoferrin, which both reduces inflammatory responses and inhibits the proliferation of pathogens [20]. Other important factors are oligosaccharides which support the development of a healthy microbiome as well as prevent pathogens from adhering to the mucosa of the infant gut and causing diarrhoea. Although the pasteurisation process destroys some of the immune properties in DHM, there are still sufficient to provide protection against viruses and bacteria and help reduce the incidence of infection [21, 22]. This reflects the findings in the infants fed DHM at ITL.

A reduction in recurrent opportunistic respiratory infections particularly in the HIV positive infants who had also had tuberculosis, could possibly be attributed to the immune properties in the breast milk. A meta-analysis of studies from 1980 to 2001 found that breastfed infants had a 72% lower risk of being hospitalised for respiratory infections [23, 24]. Tromp et al. reported that breastfeeding for six months or longer was significantly associated with a reduced risk of lower respiratory infections up to four years of age (OR: 0.71; 95% CI 0.51, 0.98) [25]. These findings supported the reduction of recurrent chest infections reported in this paper.

Evidence around the protective role of breastfeeding against developing atopic allergies and disease is conflicting [26]. This can possibly be attributed to different methodologies used and the outcomes set. Gdalevich et al. reported a protective effect of breastfeeding on atopic allergies when infants were exclusively breastfed for three months and had a family history of allergy [27], while other researchers found an increased risk of allergies [28, 29]. Many of the cases reported in this paper demonstrated an improvement in eczema once on DHM, which seems to contradict recent findings by Soto-Raminez et al. [30]. They reported that any mode of feeding which included formula or bottle-fed breast milk had an increased risk of for eczema/skin allergy in the first six years of life (PR = 1.46) as opposed to infants who were directly breastfed. The infants fed on breast milk in these case studies were bottle-fed and we noted an improvement in their condition.

What was observed was an improvement in the quality of life, relief from discomfort and distress of associated conditions. Infants grew well, the majority reached their milestones timeously and were adopted as happy well-adjusted infants or children. Many of the infants who arrived at the transition home had been cared for in hospitals or other places of care prior to admission, the major difference being that at ITL they were fed with DHM.

Making DHM more accessible to vulnerable infants within communities is challenging. Firstly, the number of breastfeeding mothers required to provide sufficient DHM to feed term infants is substantial. Human milk banking always needs to go hand-in-hand with the protection, promotion and support of breastfeeding. Using DHM to feed vulnerable infants raises the profile of the importance of breastfeeding and improves breastfeeding rates [31]. Furthermore, it encourages women to donate as they see the direct benefits of using donor milk.

Secondly, setting up a human milk bank is costly and often unaffordable in communities where they are needed most. One way of making DHM more affordable and accessible within communities is the availability of low cost, mobile, user-friendly pasteurizers. One such possibility is the PiAstra which is an innovative human milk pasteurising system which uses the flash-heating pasteurisation method, bringing the milk to 72 °C for 15 s [32]. Alternatively, commercial pasteurisers come in a variety of sizes and use the Holder method of pasteurisation which brings the milk to 62.5 °C for 30 min. Both methodologies have been shown to inactivate viral and bacterial contaminants in human milk [33].

## Conclusion

In conclusion, although there is documentation on the importance/efficacy of donor human milk for vulnerable low birthweight infants in the first few weeks/months of life, this case series has highlighted the observed beneficial effects of also using DHM for older vulnerable infants. The observational case reports highlight the value of DHM as an essential part of care for vulnerable infants and young children to ensure optimal growth, health and wellbeing. Furthermore, these observations reveal the importance of using DHM as an intervention to both alleviate suffering, improve outcomes and protect these children from unnecessary ill health and sub-optimal growth and development. Further research is needed with prospective, meticulous record keeping to further substantiate the benefits of using DHM for older vulnerable infants and children.

## Abbreviations

ARV: Antiretroviral treatment
DHM: Donor human milk
EBF: Exclusive breastfeeding
HC: Head circumference
HIV: Human immunodeficiency virus
ITL: iThemba Lethu transition home
L: Length
TB: Tuberculosis
